# Co-expression network and comparative transcriptome analysis for fiber initiation and elongation reveal genetic differences in two lines from upland cotton CCRI70 RIL population

**DOI:** 10.7717/peerj.11812

**Published:** 2021-07-21

**Authors:** Xiao Jiang, Liqiang Fan, Pengtao Li, Xianyan Zou, Zhen Zhang, Senmiao Fan, Juwu Gong, Youlu Yuan, Haihong Shang

**Affiliations:** 1 State Key Laboratory of Cotton Biology, Institute of Cotton Research, Chinese Academy of Agricultural Sciences, Anyang, Henan, China; 2 School of Agricultural Sciences, Zhengzhou University, Zhengzhou, Henan, China; 3 School of Biotechnology and Food Engineering, Anyang Institute of Technology, Anyang, Henan, China

**Keywords:** *G. hirsutum*, Fiber initiation, Fiber elongation, DEGs, RNA-seq, WGCNA

## Abstract

Upland cotton is the most widely planted for natural fiber around the world, and either lint percentage (LP) or fiber length (FL) is the crucial component tremendously affecting cotton yield and fiber quality, respectively. In this study, two lines MBZ70-053 and MBZ70-236 derived from *G. hirsutum* CCRI70 recombinant inbred line (RIL) population presenting different phenotypes in LP and FL traits were chosen to conduct RNA sequencing on ovule and fiber samples, aiming at exploring the differences of molecular and genetic mechanisms during cotton fiber initiation and elongation stages. As a result, 249/128, 369/206, 4296/1198 and 3547/2129 up-/down- regulated differentially expressed genes (DGEs) in L2 were obtained at −3, 0, 5 and 10 days post-anthesis (DPA), respectively. Seven gene expression profiles were discriminated using Short Time-series Expression Miner (STEM) analysis; seven modules and hub genes were identified using weighted gene co-expression network analysis. The DEGs were mainly enriched into energetic metabolism and accumulating as well as auxin signaling pathway in initiation and elongation stages, respectively. Meanwhile, 29 hub genes were identified as *14-3-3*ω**, *TBL35*, *GhACS*,* PME3, GAMMA-TIP*, *PUM-7*, etc., where the DEGs and hub genes revealed the genetic and molecular mechanisms and differences during cotton fiber development.

## Introduction

Cotton is one of the most important cash crops around the world, providing the main natural fiber for the textile industry. Due to its adaptability and yield, upland cotton has been the most widely cultivated *Gossypium* species, which could contribute to almost 95% production of all planted cotton in spite of presenting the relatively ordinary fiber quality (Yoo & Wendel, 2014). However, either fiber yield or quality traits of cotton are sensitive to the environment, belonging to quantitative traits controlled by multi-genes, therefore breeders focus on developing new upland cotton varieties simultaneously performing superior fiber quality and high yield. It would be not only significantly beneficial for upland cotton breeding, but also for the global textile industry (Kim & Triplett, 2001).

On the basis of previous researches on cotton, both fiber yield and quality performances are collectively determined by the four developmental stages, which could be classified into: initiation (−3 to 3 DPA), elongation (3 to 23 DPA), secondary wall biosynthesis (20 to 40DPA) and maturity (40 to 50DPA) (Basra & Malik, 1984; Kim & Triplett, 2001; Lee et al., 2006; Lee, Woodward & Chen, 2007; Haigler et al., 2012). The developmental stages of fiber initiation, elongation and secondary wall biosynthesis prevailingly affect fiber number, length and strength, respectively (Basra & Malik, 1984; Patel et al., 2020). During fiber initiation, trichome protrusion and enlargement of epidermal cells (Qin & Zhu, 2011), and fiber initiation had great impact on the number of lint fibers because the later initials always develop as fuzz fibers (Lee et al., 2006). In fiber elongation, fiber cells started primary cell wall biosynthesis alongside pectin biosynthesis genes expressed, which promoted fiber elongation by ethylene signaling pathways (Pang et al., 2010). Therefore, to explore the upland cotton agronomy traits of lint percentage (LP) and fiber length (FL), we focused on initiation and elongation stages.

Along with the rapid development of sequencing technology, reference genomes of diploid and allotetraploid *Gossypium* species have been successfully sequenced, constructed and published, which provide a solid foundation for researching on the genetic mechanisms at the genome level (Paterson et al., 2012; Li et al., 2014; Li et al., 2015; Zhang et al., 2015; Hu et al., 2019; Wang et al., 2019; Yang et al., 2019; Huang et al., 2020). Transcriptome sequencing, known as RNA-seq, provides a suitable procedure to analyze individual gene transcription and the entire transcriptome profile during various stages of fiber development. Concentrating on analysis of differential expressed genes, comparative transcriptome is an efficient tool to scan candidate genes between different samples. In the past few years, numerous studies have used comparative transcriptome analysis on cotton fiber development (Applequist, Cronn & Wendel, 2001; Gilbert et al., 2014; Yoo & Wendel, 2014; Islam et al., 2016; Li et al., 2017a; Li et al., 2017b; Lu et al., 2017; Zou et al., 2018). However, there were few studies concentrating on fiber initiation and elongation stages or using extreme materials in breeding population.

To explore the parental source of potential alleles, CCRI70 RIL population was developed. In this study, the two lines MBZ70-053 (L1, high-FL) and MBZ70-236 (L2, high-LP) derived from upland cotton RIL population, presenting excellent performances either in cotton yield or in fiber quality trait, were applied to comparative transcriptome analysis using RNA sequencing aimed at revealing the differences on a transcription level between the two lines during fiber initiation and elongation. Through DEG and WGNCA analyses, two *GAMMA-TIP, GhAcs6, Sus4, PME3* and other key candidate genes were identified, which might have great influence on cotton fiber initiation and elongation. All those provided insights and evidences for understanding molecular mechanism of cotton fiber development and differences leading to the negative correlation between quality and yield traits on transcription level that would be beneficial for upland cotton breeding.

## Materials and Methods

### Plant materials

Upland cotton hybrid CCRI70 (F_1_), the first national approved higher fiber quality hybridized upland cotton variety for utilizing heterosis between the conventional cotton varieties in China, was developed from two upland cotton cultivars sGK156 and 901-001, which performed superior yield and fiber quality, respectively. While CCRI70 showed excellent performance in fiber quality while moderate performance in lint percentage. To investigate the parental source of potential alleles and to explore the molecular and genetic mechanisms aiming at improving fiber quality and yield, we developed the CCRI70 RIL population (Zou et al., 2018; Deng et al., 2019). The CCRI70 RIL population was planted in 2015 and 2016 (Zou et al., 2018). Phenotypic data in five environments were used in this study, including Anyang of Henan Province in 2015 and 2016 (15AY and 16AY), Linqing of Shandong Province in 2015 and 2016 (15LQ and 16LQ) and Changde of Hunan Province in 2016 (16CD).

In 2017, MBZ70-053 (L1) and MBZ70-236 (l2), designated from *F*_8:9_ family of CCRI70 RIL population, were used as plant materials for conducting RNA-seq. The two lines were planted under standard field conditions in Anyang Experimental Station (Anyang, Henan, China) (Zou et al., 2018). Among the two materials, L1 showed positive extreme-parent performance in FL and negative extreme-parent in LP as well as L2 possessed positive extreme-parent performance in LP and negative extreme-parent in FL. The day of anthesis was marked as 0 DPA. According to the size of the buds and extensive field experience, the flower buds at 3 days before anthesis were recorded as −3 DPA. At −3 and 0 DPA, cotton ovules were collected from ovaries, while fiber samples were collected from bolls at 5 and 10 DPA, respectively. Both the ovule and fiber samples prepared for RNA-seq analysis were collected with three biological repeats and frozen by liquid nitrogen. For convenience, samples of L1 and L2 used in this research were recorded as L1_-3DPA, L1_0DPA, L1_5DPA, L1_10DPA, L2_-3DPA, L2_0DPA, L2_5DPA and L2_10DPA, respectively.

### Phenotypic data evaluation in multiple environments

Two RILs and two parents were planted with two replications in five environments across two years and three locations. To evaluate the phenotypic date of FL and LP, thirty mature fully-opened bolls from every plot were harvested to test fiber length using an HVI1000 (Uster Technologies, Switzerland) with HVICC Calibration in the Cotton Quality Supervision, Inspection and Testing Center, Ministry of Agriculture, Anyang, China. Briefly, after the seed cotton samples were weighed and ginned, lint percentage was evaluated.

### RNA isolation, cDNA library construction, Illumina deep sequencing and RNA-seq data analysis

Total RNAs of ovule and fiber samples were extracted by RNAprep Pure Plant Kit (Polysaccharides& Polyphenolics-rich, Tiangen, Beijing, China), and RNA degradation and contamination were checked by 1% agarose gel electrophoresis. The RNA concentration was confirmed using NanoDrop 2000 spectrophotometer (Thermo Scientific, Waltham, MA, USA). RNA purity was detected using the NanoPhotometer spectrophotometer (IMPLEN, CA, USA). The RNA integrity was confirmed using the RNA Nano 6000 Assay Kit of the Bioanalyzer 2100 system (Agilent Technologies, CA, USA). According to the manufacturer’s recommendations, an amount of 2 µg RNA per sample was used for transcriptome library construction using Illumina TruSeq™ RNA Sample Preparation Kit (Illumina, San Diego, CA, USA) Totally, 24 libraries were separately sequenced using Illumina Novaseq 6000 sequencing platform with 150 base pair (bp) paired-end (PE) raw reads (BerryGenomics Co., Ltd., Beijing, China).

Subsequently, Trimmomatic software was utilized to process all the generated raw data in Fastq format (Bolger, Lohse & Usadel, 2014). Clean data were obtained by removing reads that contained the adapter, poly-N and low-quality reads, of which the reads harbored ≥10% unidentified nucleotides (N) and >20% bases with Phred quality <5. Meanwhile, the GC percentage and Q30 were calculated to finally evaluate the quality of clean data, which were qualified for downstream analysis. HISAT2 v2.1.0 was used to build an index of reference genome (Pertea et al., 2016), and the sequence alignment was conducted referring to the *G. hirsutum* genomes (Wang et al., 2019) with default parameters, where the reference genome was available at the website http://cotton.hzau.edu.cn/EN/download.php and the CottonGen database (https://www.cottongen.org/). Then the fragments per kilobase of exon per million reads (FPKM) values of genes were quantized by StringTie v1.3.5 (Pertea et al., 2015), which were subjected to Pearson correlation coefficient (PCC) for revealing the correlation coefficients between samples. As to the correlation coefficients less than 0.8 among the three biological repeats, the samples would be removed from the dataset.

Furthermore, to identify the genetic differences between the two lines, we employed Samtools v1.4 software to summarize the genotypic data (Li et al., 2009; Li, 2011) and SNPEff program to annotated the genotypic variants distribution on the reference genome (Wang et al., 2019) with default parameter (Cingolani et al., 2012).

### Differentially expressed gene analysis

Based on the count number of each gene, the DESeq2 R package was employed for identifying differentially expressed genes (DEGs), of which the screening criterion were FDR value <0.05, and log_2_ Fold-Change value >1 or <−1 between each pairwise comparison (Love, Huber & Anders, 2014). The DEGs were identified through vertical and horizontal comparisons, i.e., at the same developmental stage between the two lines and in the same line between different stages.

To explore the temporal expression profiles of DEGs during the fiber development, Short Time-series Expression Miner (STEM) was conducted to analyze the DEGs expression patterns in two lines (Ernst, Nau & Bar-Joseph, 2005). The enrichment analysis of Kyoto Encyclopedia of Genes and Genomes (KEGG) and Gene Ontology (GO) analysis were based on KOBAS 3.0 software, BLASTX, and GO databases (http://archive.geneontology.org/latest-lite/) (Altschul et al., 1990; Wu et al., 2006; Xie et al., 2011), respectively.

### Scanning DEGs in quantitative trait locis

To identify the potential candidate alleles in CCRI70 RIL population, we compared the DEGs with previous quantitative trait loci (QTL) result (Deng et al., 2019). Mapping the simple sequence repeat (SSR) loci sequences of LP and FL stable QTLs to the new reference genome using bowtie software (Langmead et al., 2009). Scanning the DEGs of −3 and 0 DPA in the physical confidence intervals of LP QTLs while comparing the DEGs of 5 and 10 DPA in FL QTLs.

### Hub genes identification and co-expression networks construction

WGCNA (weighted gene co-expression network analysis) R package was used for identifying modules and hub (or highly correlated) genes that highly associated with fiber initiation and elongation (Langfelder & Horvath, 2008). The topology overlap matrix was built with hierarchical clustering method, and the dynamic tree cut and merged into modules. Among the modules, what containing a coefficient (>0.6) with each sample were identified as key modules and organized for co-expression networks construction. In WGCNA, K _ME_ was a value to describe the eigengene connectivity. In this study, the DEGs with the highest K _ME_ values in each key module were identified as hub genes (Pei, Chen & Zhang, 2017). The top 200 pairs of network connections stored in the edges files by weight value were selected to build interaction networks within DEGs, and the hub genes were selected by the basis of module membership (K_ME_) values, of which the interaction networks were drawn by Cytoscape 3.7.1 (Shannon et al., 2003).

### Hub genes and DEGs expression pattern validation

To validate the expression pattern, we performed qRT-PCR on hub genes and selected DEGs. The samples of cDNA were synthesized from 1µg of total RNA by using *TransScript^®^* II All-in-One First-Strand cDNA Synthesis SuperMix for qPCR (TransGen Biotech co., ltd, China). Real-time PCR was performed by using *TransStart^®^ Taq* DNA Polymerase (TransGen Biotech co., ltd, China) and LightCycler^®^ 480 II Real-time PCR instrument (Roche, Basel, Switzerland). The specific primers for qRT-PCR were designed referring to qPrimerDB (https://biodb.swu.edu.cn/qprimerdb) (Lu et al., 2018). Gene expression levels were calculated according to the 2^−ΔΔCt^ method with three biological (Livak & Schmittgen, 2001).

## Results

### Phenotypic data analysis of the two lines

**Figure 1:**
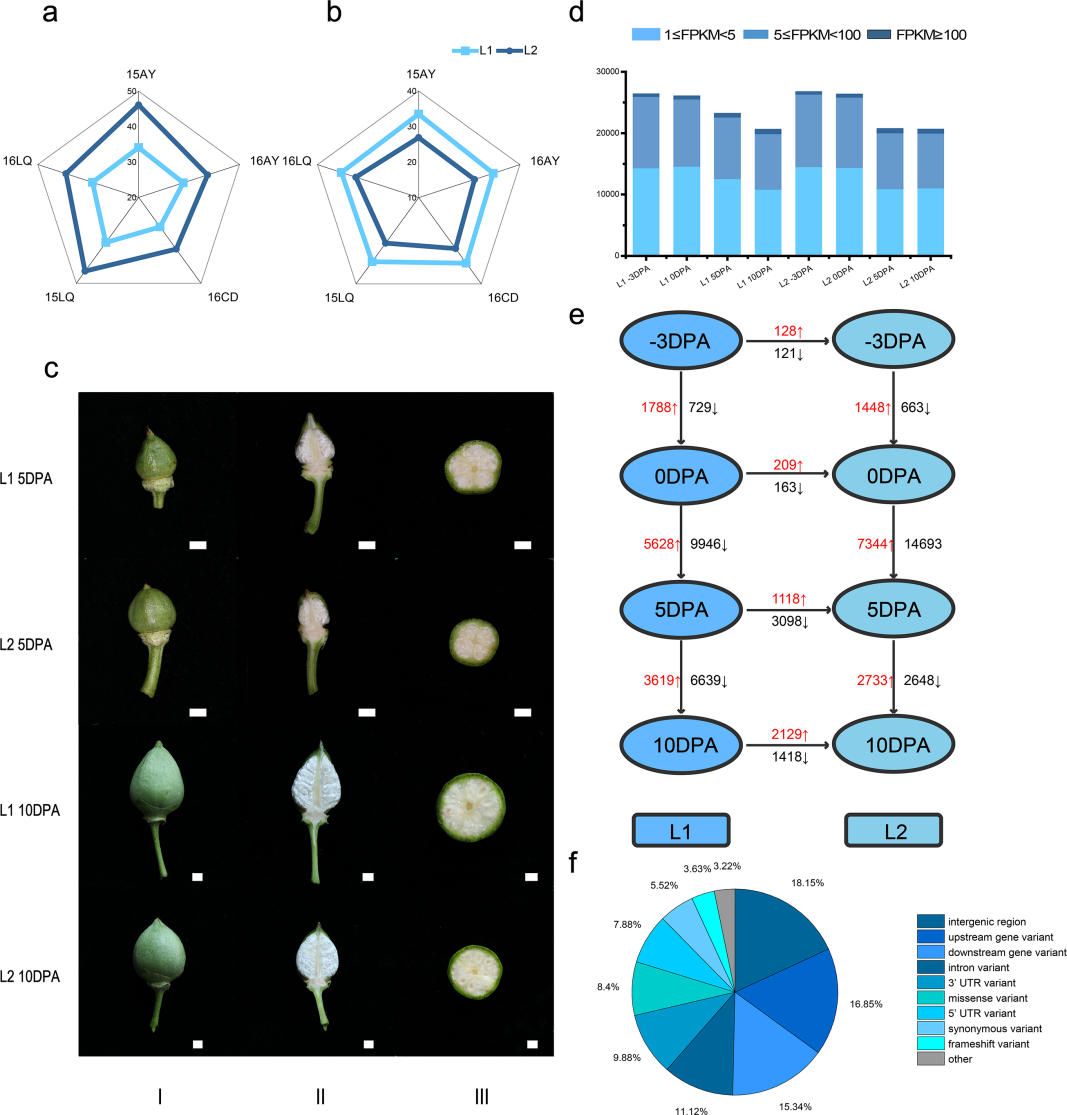
Phenotypic data, statistics for transcript levels at each development stage and vertical of L1 relative to L2 and horizontal comparisons of DEGs. (A) Phenotypic data of lint percentage (LP) trait in five environments of L1 and L2; (B) Phenotypic data of fiber length (FL) trait in five environments of L1 and L2; (C) Photos of bolls of L1 and L2 at 5 and 10 DPA, column I, II and III refer to front photo, longitudinal cut and crosscut of cotton bolls, respectively; (D) Statistics for transcript levels of each sample at each development stage, the numbers of expressed genes were divided by 0.5 <FPKM <5, 5 <FPKM <100 and 100 < FPKM. (E) Vertical and horizontal comparisons showed the DEGs in the same developmental stage between the two lines (L2 relative to L1) and in the same line between different stages. The numbers of upregulated and downregulated genes were marked in red and black, respectively. (F) Variant type distribution.

In the five environments, Lint percentage (Fig. 1A) in L1 and L2 were 34.40% and 42.86% as well as the fiber length were 32.60 mm and 27.51 mm (Fig. 1B), respectively, which indicated that L1 had longer FL while L2 had higher LP (Table S1).

### Transcriptome sequencing analysis and correlation of replicate samples

To reveal gene expression during fiber development, we conducted transcriptome sequencing (RNA-seq) on ovule samples at −3 and 0 DPA as well as fiber samples at 5 and 10 DPA (Fig. 1C). A total of 941.90 million clean reads were obtained from 24 libraries with an average of 39.24 million reads per sample. Meanwhile, 91.16% to 95.09% of the Q30 was calculated with an average of 92.97%, while 43.02% to 51.27% of the GC content range was calculated with an average of 45.27%, which indicated the reliability of RNA-seq data (Table S2 and Fig. S1). The raw data had been submitted to National Genomics Data Center (accession number CRA002982).

Based on FPKM, genes with FPKM value not less than 1 were considered as expressed in this study and the Pearson correlation coefficient was conducted on the 24 samples (Table S3). Consequently, at −3, 0, 5 and 10 DPA, 31456, 31330, 27737 and 25120 expressed genes were identified in L1, respectively. Meanwhile, 31867, 31494, 25215 and 25146 genes were expressed in the L2. The percentages of expressed genes with FPKM values of 1–5, 5–100 and >100 were 54.9%, 43.7% and 1.4%, respectively (Fig. 1D).

### Differentially expressed genes analysis

To identify the significantly differentially expressed expressed genes during fiber initiation and elongation, we used DESeq2 R package between each pairwise comparison. Through vertical (Table S4) and horizontal (Table S5) comparisons, after removing the duplicate genes, 30352 unique DEGs (Fig. 1C, Table S6) were identified during cotton fiber development.

At 0 DPA, 369 DEGs were identified, including 206 up-regulated (log_2_(FC) >1) and 163 down-regulated (log_2_(FC) <−1), of which the representative DEGs showed high —log_2_(FC)— between L1 and L2 were listed in Table S7. Among up-regulated genes, *Ghir_A05G006080* was enriched in the carbon metabolism pathway and annotated as *NP-GAPDH*, which was involved in catalyzing the oxidation of Ga3P to 3-phosphoglycerate (Valverde et al., 2005) and was important in fruit development and energetic metabolism (Rius et al., 2006); *Ghir_D08G011800* was annotated as UDP-glycosyltransferase superfamily protein and participates in starch biosynthetic process presenting a direct influence on starch glycan composition (Ortiz-Marchena et al., 2014), which might be relevant to accumulating and mobilizing sugars process. Among the down-regulated genes, *Ghir_D13G006000* was annotated as alpha-galactosidase 2*,* enriched in the galactose metabolism pathway, related with cell wall loosening during cell growth in *Arabidopsis* and barley, it was involved in lengthening the polymers occurring in the wall, upon secretion, or for binding of the XyGs to cellulose (Peña et al., 2004) and was specifically localized in the cell wall (Chrost et al., 2007); *Ghir_D06G012250* was annotated as disproportionating enzyme 2 and enriched in starch and sucrose metabolism pathway, which could utilize maltose as glucosyl donor and glycogen as acceptor releasing the other hexosyl unit as free glucose that then are further metabolized by the cellular central carbon metabolism (Andersson & Rådström, 2002); Le (Breton et al., 2005; Smirnova et al., 2017).

At 5 DPA, there were 1198 up-regulated and 3098 down-regulated DEGs between L1 and L2. 156 representative ones with high expression (FPKM >5) or high —log_2_(FC)— (>2) are shown in Table S8. Based on KEGG enrichment analysis on the up-regulated DEGs, *Ghir_A13G021680*, *Ghir_A11G013660*, *Ghir_A11G006910*, *Ghir_D11G007650*, *Ghir_A11G025370*, and *Ghir_D05G001330* were enriched into SNARE interactions of vesicular transport pathway, which was participated in endoplasmic reticulum to Golgi vesicle-mediated transport and membrane fusion (Schiller et al., 2012; Bolaños Villegas, Guo & Jauh, 2015; Sharma et al., 2017). In addition, the down-regulated DEGs were identified to be mainly enriched in plant hormone signal transduction, starch and sucrose metabolism and metabolic pathways, suggesting that in the line with superior fiber quality these pathways play important roles during fiber elongation. *Ghir_D05G014410* was annotated as pectin methylesterase 3 (*PME3*) which was ubiquitously expressed, particularly in vascular tissues and had influence on degree of methylesterification of galacturonic acids. The reaction of demethylesterification decreased the extracellular pH to increase the hydrolytic enzyme activities of enzymes such as poly-galacturonic acid and several pectin enzyme cleavage enzymes (Wen, Zhu & Hawes, 1999), when pectin was subject to substantial degradation leading to cell wall structure relaxation and enhancing the growth of cell tips (Catoire et al., 1998; Li et al., 2016). Meanwhile, *PME3* was also reported affecting the number of adventitious roots (Guénin et al., 2011); *Ghir_A13G020210* was annotated as *sucrose synthase 4 (Sus 4)*, where *Sus* was demonstrated to be critically important for cotton fiber initiation and elongation (Ruan, Llewellyn & Furbank, 2003). *Ghir_D07G008950*, *Ghir_D10G022670*, *Ghir_A05G005960* and *Ghir_A10G020870* were enriched into phenylalanine metabolism pathway, which was involved in lignin polymer producing and secondary cell wall construction (Barros et al., 2016; Zhou et al., 2017; Vanholme et al., 2019). *Ghir_D05G003750* was annotated as an auxin-responsive factor 7 (*ARF7*), which was required for leaf expansion and/or lateral root induction (Wilmoth et al., 2005). There were also some other transcription factors or genes associated with or response to auxin, such as *Ghir_A01G010000* (*ARF5*), *Ghir_D09G022910* (*ATAUX2-11*), *Ghir_D03G003390* (IAA9), *Ghir_D05G022030* (*IAA9*) and *Ghir_D12G011080* (*SAUR36*) (Aspuria et al., 2002; Fujita et al., 2012; Hou, Wu & Gan, 2013; Stamm & Kumar, 2013; Krogan et al., 2016) suggesting that auxin signaling pathway is involved in fiber elongation phase in high-FL line.

### Genotypic variants analysis

To explore the genetic differences between the two lines, we employed SNPEff program to analyze the genotypic variants distribution on the genome A total of 239493 variants were identified referring to TM-1 (Wang et al., 2019), and 40522 genes were involved, where 20181 of them were DEGs.

Among the variants, 20128 (8.40%) arisen as missense variant, 8685 frame shift mutation (3.63%) occurred, 739 variants (0.31%) led to transcription termination and 493 variants (0.21%) occurred losing of stop codon of the transcripts. These variants had of significance impact on the gene functions. In addition, 19760 and 23666 variants were identified at 5′  and 3′  UTR region, respectively, which might have influence on regulation of gene transcription (Table S9, Fig. 1F). Comparing to the DEGs in different developmental stages, 14146 variants were identified in 2662 expressed DEGs (Fig. S4). In the 157 unique significantly up or down regulated DEGs in 5 and 10 DPA (Tables S7 and S8), 652 variants were identified and 78 performed differently in 26 DEGs between two RILs. Due to insertion, *Ghir_A05G005960* occurred frame shift variant. For the SNP variant, transcription termination was occurred in *Ghir_D07G024380* and there were protein sequence mutations in 11 DEGs. There were three variants located (*Ghir_A05G006080*, *Ghir_A04G013900* and *Ghir_A10G010640*) in splice region and one identified on splice acceptor (*Ghir_D03G003390*), which might lead to alternative splicing. Those variants had significant impact on the protein sequence and functions of DEGs. Besides, 25 variants were located 5′  or 3′  UTR and 15 variants were identified at upstream or downstream that might have effects on the regulation of gene transcription.

### Temporal gene expression patterns analysis

**Figure 2:**
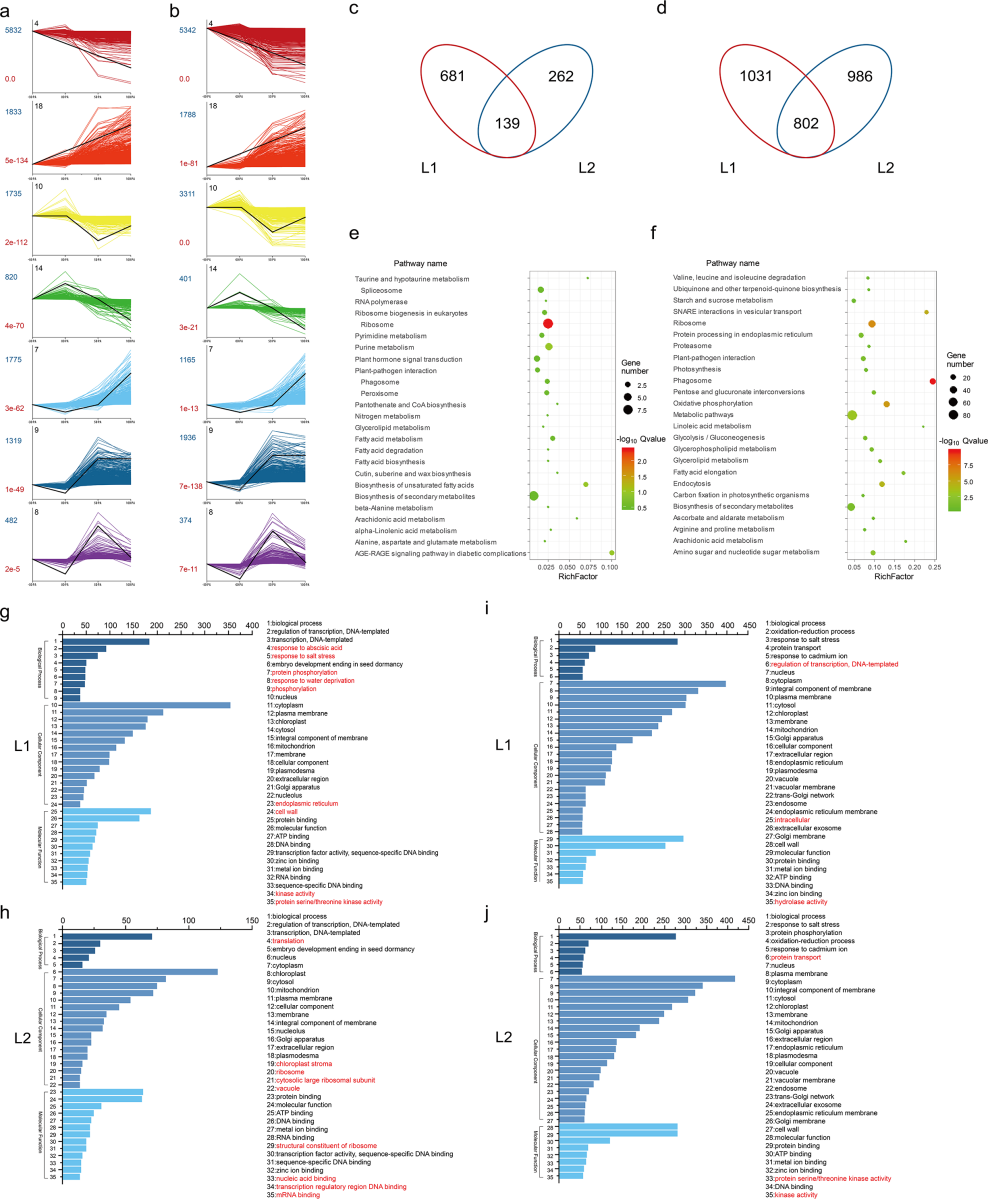
DEGs analysis. (A) Different gene expression profiles in L1 and L2. Each profile presents a gene expression trend. The profile ID, gene number and *P*-value were marked in black, darkblue and darkred, respectively; (B) Venn diagram showed the same and different genes between L1 and L2 in profile 14 and 18, respectively. (C) Kyoto Encyclopedia of Genes and Genomes (KEGG) pathways analysis of the common genes between L1 and L2 in profile 14 and 18. The size and color of bubbles presented the gene number enriched in the pathway and the size of Q-value, respectively. (D) Gene ontology (GO) enrichment analysis of the different genes between L1 and L2 in profile 14 and 18, and the top 35 terms of GO enrichment for 681, 262, 1031 and 986 genes unique to L1 and L2 in profile 14 and 18, respectively. The different terms were marked in red.

To identify the temporal expression profiles, we performed STEM analysis using all the DEGs. 17180 and 17585 DEGs were classified and organized into seven expression profiles with e-values less than 0.001 in L1 (Fig. 2A) and L2 (Fig. 2B), of which the genes in the same profile performed similar expression patterns during fiber development (Table S10). Associated with the fiber development, we focused on the DEGs in profile 14 and 18. In profile 14, the expression had high expression in -3 and 0 DPA, and then was decreasing in fiber samples. While the DEGs in profile 18 had low expression in ovule samples and had a increasing trend in 5 and 10 DPA. The trends of profile 14 and 18 fitted with fiber developmental stages of initiation and elongation, respectively. Profile 14 contained 820 genes in L1 and 401 in L2, while profile 18 identified 1833 genes in L1 and 1788 in L2. Venn diagram was utilized to visualize the gene comparison (Fig. 2C). A total of 681 and 262 DEGs were identified in profile 14 of L1 and L2, with 139 genes expressed in common. Similarly, in profile 18, 1031 and 986 genes were expressed differentially in L1 and L2, while 802 was in common (Fig. 2D).

To investigate the pathways of the common DEGs of the profile 14 and 18, we employed KEGG enrichment analysis (Table S11) and visualized the results with bubble graph. The common DEGs in profile 14 were mainly enriched in the pathways of ribosome, AGE-RAGE signaling pathway, biosynthesis of unsaturated fatty acids and fatty acid metabolism (Fig. 2E). Meanwhile, they were mainly enriched in metabolic pathways such as phagosome, biosynthesis of secondary metabolites, starch and sucrose metabolism and oxidative phosphorylation in profile 18 (Fig. 2F).

Simultaneously, to further explore the specific DEGs in the two lines of profile 14 and 18, we performed GO enrichment analysis and categorized into 35 most frequent GO terms based on biological process, cellular component and molecular function (Table S11). Compared to L1, the specific DEGs of profile 14 in L2 were annotated to the GO terms of GO:0003735 structural constituent of ribosome, GO:0006412 translation, GO:0044212 transcription regulatory region DNA binding, GO:0003676 nucleic acid binding, GO:0009570 chloroplast stroma, GO:0005840 ribosome, GO:0022625 cytosolic large ribosomal subunit, GO:0005773 vacuole and GO:0003729 mRNA binding (Figs. 2G and 2H). As for the GO terms of profile 18 in L1, there were three different enrichment terms compared to those in L2, such as GO:0005622 intracellular, GO:0016787 hydrolase activity and GO:0006355 regulation of transcription, DNA-templated. The GO enrichment analysis results suggested that transcription factors play the different roles between the two RILs during the fiber development (Figs. 2I and 2J).

### Gene co-expression network analysis and identification of hub genes in correlation networks

To broaden the further insight into the relationship between gene expression and fiber development as well as to identify genes associated with LP and FL, we constructed the co-expression networks for the DEGs of ovule (546 genes) and fiber (6976 genes) samples and analyzed through weighted gene co-expression network analysis.

**Figure 3:**
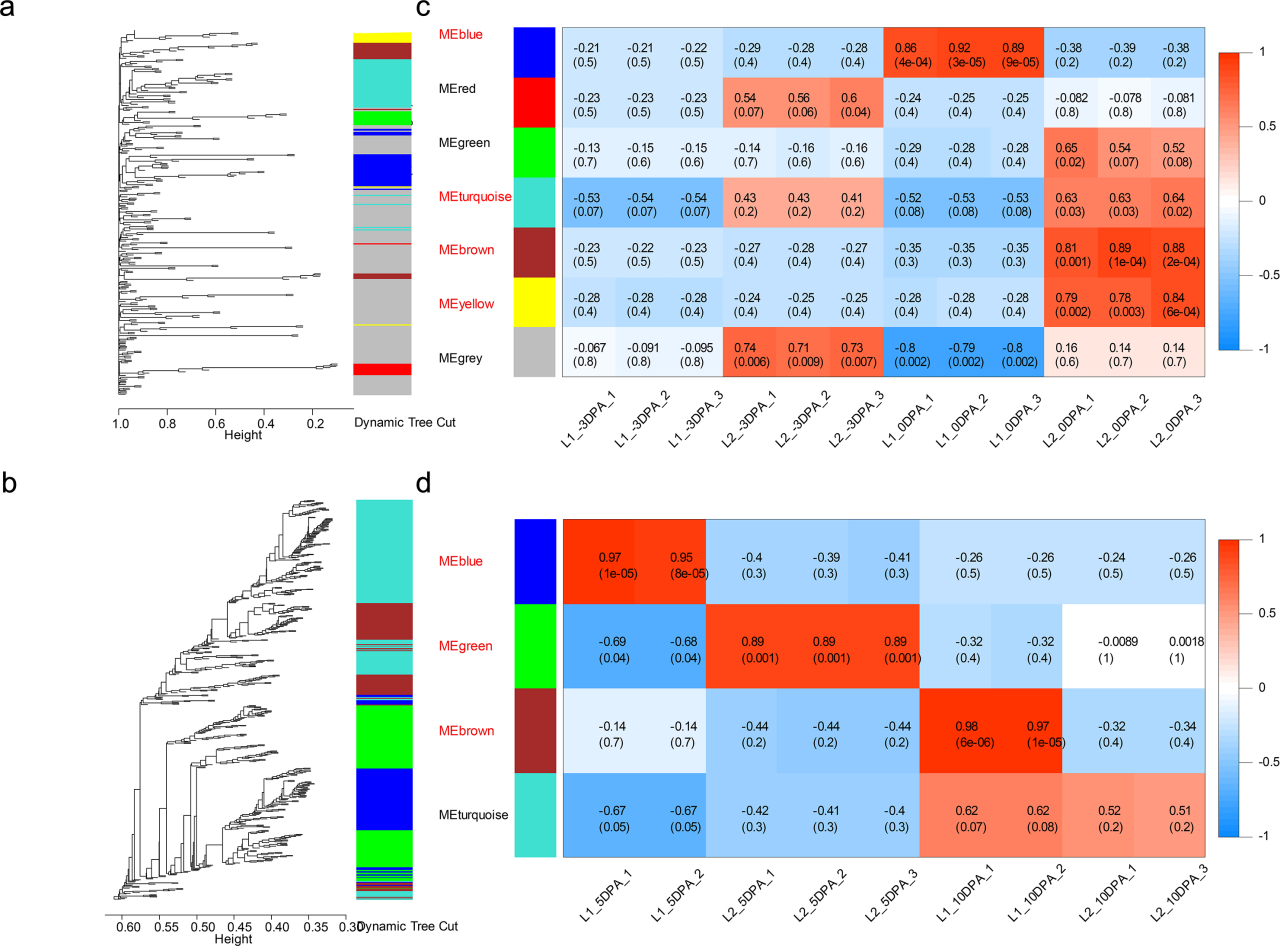
Weighted gene co-expression network analysis of DEGs at four developmental stages. Hierarchical dendrogram showing co-expression modules in ovule (A) and fiber (B) samples identified by WGCNA. Each leaf in the tree represents one gene. The major tree was divided into 11 modules in total, where seven modules were classified in ovule (C) and four in fiber (D) samples.

The dynamic tree cut and merged analogous expression patterns into modules (Figs. 3A and 3B). Finally, seven modules and four modules were identified in ovule and fiber samples (Table S12), respectively (Figs. 3C and 3D). Among them, seven key modules were identified. In ovule samples, ovule yellow, brown, turquoise and blue modules were specifically associated with initiation stage of the high-LP line. While fiber brown, blue and green modules were specifically associated with elongation stage of the high-FL line.

**Table 1:** Candidate hub genes in modules.

**Gene Name**	**KME**	**ArabidopsisID**	**Description in*Arabidopsis thaliana***
**Fiber brown module**			
*Ghir_D05G023530*	0.99	*AT1G77780*	Glycosyl hydrolase superfamily protein
*Ghir_A08G023930*	0.98	*AT1G65450*	HXXXD-type acyl-transferase family protein
*Ghir_D13G004870*	0.98		
*Ghir_D11G035770*	0.98	*AT2G36830*	gamma tonoplast intrinsic protein
*Ghir_A11G034930*	0.98	*AT2G36830*	gamma tonoplast intrinsic protein
**Fiber blue module**			
*Ghir_D08G021430*	0.99	*AT2G27040*	Argonaute family protein
*Ghir_D03G003280*	0.99	*AT5G65700*	Leucine-rich receptor-like protein kinase family protein
*Ghir_D07G014800*	0.99	*AT1G01300*	Eukaryotic aspartyl protease family protein
*Ghir_D05G019650*	0.98	*AT5G42800*	dihydroflavonol 4-reductase
*Ghir_A07G015620*	0.98	*AT2G45290*	Transketolase
**Fiber green module**			
*Ghir_A05G020170*	0.95	*AT5G42655*	Disease resistance-responsive (dirigent-like protein) family protein
*Ghir_D05G019950*	0.95	*AT1G45170*	outer envelope pore 24B-like protein
*Ghir_D11G004790*	0.95	*AT2G18170*	MAP kinase 7
*Ghir_A03G022510*	0.94	*AT1G29500*	SAUR-like auxin-responsive protein family
**Ovule yellow module**			
*Ghir_D13G021030*	0.98		
*Ghir_D05G022870*	0.97	*AT5G64260*	EXORDIUM like 2
*Ghir_A04G002270*	0.95	*AT2G04890*	SCARECROW-like 21
*Ghir_A12G024510*	0.95	*AT4G11280*	1-aminocyclopropane-1-carboxylic acid (acc) synthase 6
**Ovule brown module**			
*Ghir_A03G022700*	0.98	*AT2G17200*	ubiquitin family protein
*Ghir_D08G021550*	0.98	*AT2G28380*	dsRNA-binding protein 2
*Ghir_D05G012040*	0.96	*AT1G80830*	natural resistance-associated macrophage protein 1
**Ovule turquoise module**			
*Ghir_A01G001190*	0.97	*AT1G78300*	general regulatory factor 2
*Ghir_A07G020020*	0.96	*AT5G27260*	Myb/SANT-like DNA-binding domain protein
*Ghir_A09G017620*	0.96	*AT5G01620*	TRICHOME BIREFRINGENCE-LIKE 35
*Ghir_A05G042810*	0.94	*AT5G54130*	Calcium-binding endonuclease/exonuclease/phosphatase family
**Ovule blue module**			
*Ghir_A08G021080*	0.98	*AT2G30090*	Acyl-CoA N-acyltransferases (NAT) superfamily protein
*Ghir_A06G019930*	0.96	*AT3G15820*	phosphatidic acid phosphatase-related / PAP2-related
*Ghir_A11G031900*	0.96	*AT2G44260*	Plant protein of unknown function (DUF946)
*Ghir_D13G001750*	0.94	*AT3G11210*	SGNH hydrolase-type esterase superfamily protein

**Figure 4:**
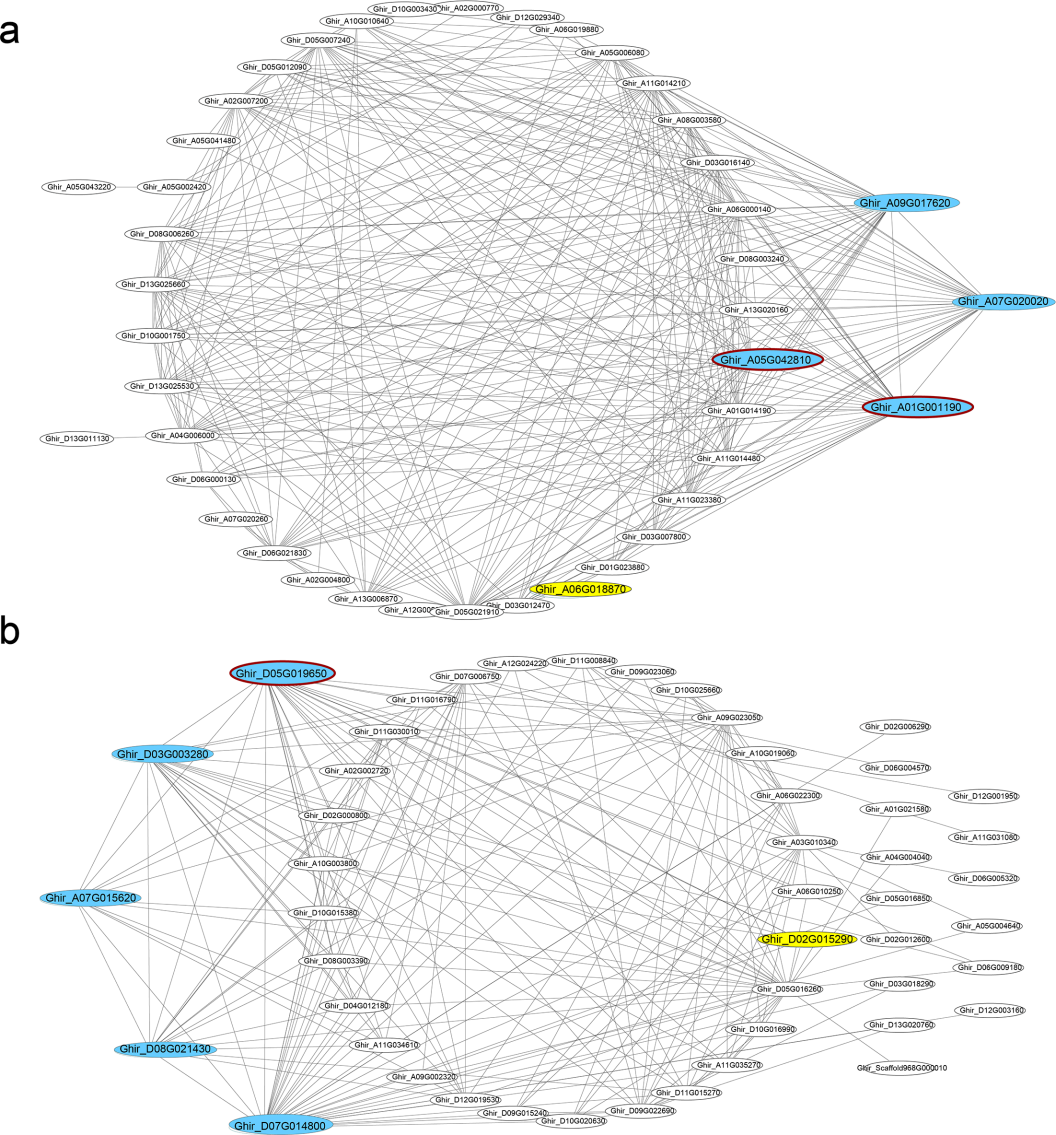
Co-expression networks. Hub genes and transcription factors of (A) ovule turquoise module and (B) fiber blue module, which are highlighted in cyan and yellow, respectively. The hub genes circled dark red shared genotypic differences.

In seven key modules and according to K_ME_, 29 DEGs genes with the highest eigengene connectivity in each module were identified as hub genes (Table 1). All the hub genes performed the K_ME_ values greater than 0.94. The hub genes in ovule yellow module encoding EXORDIUM like 2 protein, SCARECROW-like 21 protein and 1-aminocyclopropane-1-carboxylic acid (acc) synthase 6 (ACS6) protein, where *acs6* was identified involved in cell division and ethylene biosynthesis (Luo et al., 2014; Yin et al., 2019). *GhACS* and ethylene were playing important roles in cotton fiber development (Wang et al., 2007). Hub genes enriched in ovule brown module, encoded ubiquitin family protein, dsRNA-binding protein 2 and natural resistance-associated macrophage protein 1. Additionally, in ovule turquoise module (Fig. 4A), the hub genes were annotated as general regulatory factor 2 (GRF2), myb/SANT-like DNA-binding domain protein, TRICHOME BIREFRINGENCE-LIKE 35 (TBL35) protein and Calcium-binding endonuclease/exonuclease/phosphatase family protein, of which *Ghir_A09G017620* annotated as *TBL35*, may participate in xylan acetylation (Yuan et al., 2016). *Ghir_A01G001190* was annotated as G-box binding factor GF14 omega encoding a 14 − 3 − 3 protein, which was reported to be located at the regions of the plant that comprise dividing cells and involved in plant cell cycle (Sorrell et al., 2003).

In the modules of fiber tissues at 5 and 10 DPA, hub genes were critically associated with the fiber elongation stage. The hub genes in blue module at 5 DPA encoded an argonaute family protein, a leucine-rich receptor-like protein kinase family protein, a eukaryotic aspartyl protease family protein, dihydroflavonol 4-reductase and transketolase, which was involved in vascular development (Qian et al., 2018). Similarly, the green module contained the hub genes encoding a disease resistance-responsive (dirigent-like protein) family protein, an outer envelope pore 24B-like protein, a SAUR-like auxin-responsive protein and a MAP kinase 7. At 10 DPA, the hub genes in brown module were annotated with a glycosyl hydrolase superfamily protein, a HXXXD-type acyl-transferase family protein and two gamma tonoplast intrinsic proteins. Among the hub genes in fiber blue (Fig. 4B) and brown modules, *Ghir_D11G035770* and *Ghir_A11G034930* were both identified as hub genes in fiber brown module and were annotated as gamma tonoplast intrinsic protein (GAMMA-TIP1), which is mainly expressed in vessel-flanking cells of vascular bundles (Beebo et al., 2009) and confirmed to mediate unbalanced water content in leaves (Zhu et al., 2014). *Gh γTIP1* during fiber cell elongation played important roles in supporting the rapid influx of water into vacuoles during cotton fiber cell expansion (Liu et al., 2008). Besides, *Ghir_A01G001290*, sharing high eigengene connectivity, was annotated as APUM-7 translation factor, and *Ghir_A01G005740* encoded a domain of unknown function (DUF1218) family protein, whose homologous gene was knocked-out showing a reduction in total xylem (Ubeda-Tomas et al., 2007). The protein containing DUF1218 domain played important role in xylogenesis and/or secondary cell wall formation (Mewalal et al., 2016).

Scanning the genotypic variants in the hub genes, 161 variants were identified in the transcripts of hub genes and 51 variants performed differently between the two RILs. Due to the variants, transcription termination of *Ghir_A05G042810* and *Ghir_D13G001750* occurred in L2 and frame shift mutation of *Ghir_A01G001190* occurred in L1 with one nucleotide deletion, which might have huge impact on the protein sequences. In addition, one SNP variant located on the intron of *Ghir_A05G042810* that might lead to alternative splicing and changing the protein sequence. Causing by SNP variant, missense variants occurred in another five hub genes that changed the protein primary structure (*Ghir_A06G019930*, *Ghir_D05G019650*, *Ghir_D05G019950*, *Ghir_D05G022870* and *Ghir_A05G042810*). 14 variants were identified at 5′  or 3′  UTR and 16 were located at downstream that might have effects on the regulation of gene transcription.

### qRT-PCR expression pattern validation

**Figure 5:**
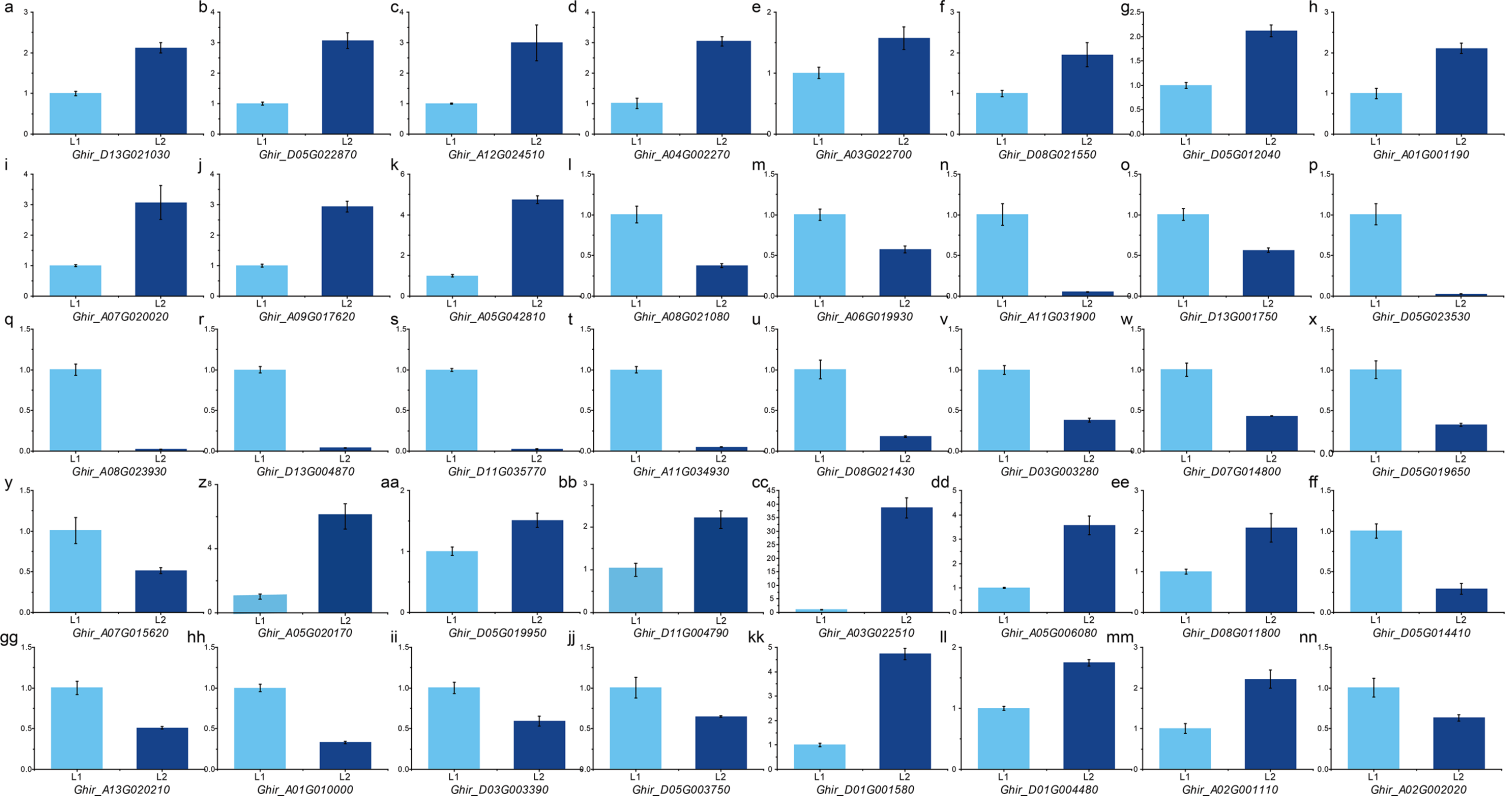
qPCR analysis of hub genes and key DEGs. qPCR results of the hub genes in ovule yellow (A–D), brown (E–G), turquoise (H–K) and blue (L–O) modules as well as the hub genes in fiber brown (P–T), blue (U–Y) and green (Z–CC) modules. And the key DEGs during fiber development (BB–JJ) and the DEGs in QTLs (KK–NN).

To validate the expression pattern, we performed qRT-PCR on important DEGs and hub genes using the primers according to qPrimerDB (Table S13). The expression pattern validation result of the 29 hub genes from ovule yellow (Figs. 5A–5D), ovule brown (Figs. 5E–5G), ovule turquoise (Figs. 5H–5K), ovule blue (5L– 5O), fiber brown (Figs. 5P–5T), fiber blue (Figs. 5U–5Y) and fiber green (Figs. 5Z–5CC) modules was similar to RNA-seq result. While the 11 key DEGs performed the similar result (Figs. 5 and 5D–5NN) (Table S14). Those results indicated the RNA-seq result is reliable.

## Discussion

### Transcriptome sequencing of two extrame-parent RILs provided new insight for exploring the expression profile in fiber initiation and elongation stages

To explore the genetic and molecular mechanisms during fiber initiation and elongation, we selected two extreme RILs form CCRI70 RIL population and conducted RNA-seq. In recent years, RNA-seq has been largely applied into cotton fiber development researches (Gilbert et al., 2014; Yoo & Wendel, 2014; Islam et al., 2016; Li et al., 2017b; Li et al., 2017a; Lu et al., 2017; Zou et al., 2019) as well as under biotic (Xu et al., 2011; Zhang et al., 2017) and abiotic stresses (Zhang et al., 2016a). Referring to the nearly studies of fiber development using RNA-seq, elongation and secondary wall biosynthesis were more often concentrated in, while the initiation stage was seldom focused on. In our work, ovule and fiber samples collected during cotton fiber initiation and elongation were conducted RNA-seq, which provided plenty of valuable data for investigating and revealing the differences of genetic and molecular mechanisms during initiation and elongation on mRNA level. CCRI70 is a breeding hybrid with excellent performance in fiber length and moderate performance in lint percentage. The two extreme RILs provide an ideal model to investigate the candidate genes coming from and the differences in initiation and elongation stages. What’s more, what good alleles related to lint percentage and fiber length were found would also benefit upland cotton breeding to improve yield and fiber quality simultaneously. In our study, 941.90 million clean reads were obtained from 24 libraries, with an average of 39.24 million per sample. Among the 24 libraries, the GC% and Q30 were 45.27% and 92.97% on average, respectively, which indicates that the quality of the RNA-seq data is reliable. However, three samples in the third biological replicate showed low correlation (<0.8), which might be affected by environmental and other factors. That provided ideal and fine basis for exploring the transcriptional differences during fiber initiation and elongation stages, which prevailingly affect the lint percentage and fiber length traits, respectively.

### DEGs revealing the transcriptional differences in initiation and elongation phases

Due to the initiation and elongation affected the number and length of fibers, which had influences on LP and FL traits, respectively. DEGs of each period were obtained and used for identifying the candidate genes that would reveal the genetic basis of fiber development and provide an insight into the molecular mechanism for the negative correlation between quality and yield traits. At fiber initiation stage, up-regulated genes in high-LP line L2 were mainly enriched in pentose and glucuronate interconversions, carbon metabolism, biosynthesis of secondary metabolites and metabolic pathways. *Ghir_A05G006080* (Fig. 5DD) might play the role like *NP-GAPDH*, a cytosolic non-phosphorylating NADP-dependent GAPDH that catalyzes the oxidation of Ga3P to 3-phosphoglycerate (Valverde et al., 2005; Rius et al., 2006). *Ghir_D08G011800* (Fig. 5EE) might be involved in starch biosynthetic process that had a direct influence on starch glycan composition (Ortiz-Marchena et al., 2014). At 0 DPA, the up-regulated genes were mainly enriched in the energetic metabolism and accumulating as well as mobilizing sugars process, implying that energetic metabolism and sugar transport may participate in fiber initiation and have effect on the number of cotton fiber. Among the up-regulated genes in high-FL line L1 at 5 DPA, *Ghir_D05G014410* (Fig. 5FF), annotated as *PME3*, had influence on degree of methylesterification of galacturonic acids (Wen, Zhu & Hawes, 1999; Guénin et al., 2011). Pectin was subject to substantial degradation leading to cell wall structure relaxation and enhancing the growth of cell tips (Catoire et al., 1998; Li et al., 2016). During cotton fiber development, *PME* played significant physiological role by influencing the chemical properties of pectin (Liu, Talbot & Llewellyn, 2013). A *sucrose synthase 4* (*Sus 4*), *Ghir_A13G020210* (Fig. 5GG), was specifically expressed in L1 with high FPKM, where it played a major role in metabolic regulation and sugar signaling, and silencing *Sus* expression led to a fiberless seed phenotype. *Sus* was demonstrated to be significantly important for cotton fiber development, and suppression of sucrose synthase gene expression repressed cotton fiber cell initiation, elongation, and seed development (Ruan, Llewellyn & Furbank, 2003). In addition, *Ghir_A01G010000* (Fig. 5HH), *Ghir_D03G003390* (Fig. 5II), *Ghir_D05G003750* (Fig. 5JJ) and some other genes were annotated as transcription factors or genes related to or responding to auxin demonstrated that auxin regulate fiber development and auxin signaling was shown to be important for fiber initiation and elongation (Samuel (Yang et al., 2006; Gou et al., 2007; Liu et al., 2012; Wang et al., 2013; Zhang et al., 2016b). Overexpressing *iaaM*, critically important for auxin biosynthesis, led to enhanced initiation and increased fiber length (Zhang et al., 2011). The DEGs result suggested that *Sus4, PME3* and auxin signaling pathway play important roles in fiber elongation stage. In the two materials, the DEGs were identified and enriched into energetic metabolism and sugar transport pathway in initiation stage, while the DEGs were enriched into auxin signaling pathway in rapid elongation stage.

In addition, comparing the DEGs with CCRI70 previous QTL result, 14 DEGs were located in LP or FL QTLs (Deng et al., 2019). Among them, *Ghir_D01G001580* (Fig. 5KK) and *Ghir_D01G004480* (Fig. 5LL) were up-regulated in L2 at 0 DPA and detected in LP QTL. *Ghir_D01G001580* was annotated as ATXR-2 that was involved in cellular dedifferentiation (Lee, Park & Seo, 2017). ATXR2-ARF-LBD axis was key for the epigenetic regulation of callus formation in *Arabidopsis*. *Ghir_D01G004480* was annotated as *Ku70* that was involved in repair of DNA double-stranded breaks and telomere regulation (Tamura et al., 2002) demonstrated to be required for the maintenance of chromosome stability and normal developmental growth in rice (Hong et al., 2010). Besides, *Ghir_A02G001110* (Fig. 5MM) and *Ghir_A02G002020* (Fig. 5NN) were identified in FL stable QTL. *Ghir_A02G002020* was annotated as xyloglucan endotransglucosylase/hydrolases 16 (*XTH16)* and had a higher expression in L1 at 10 DPA. XTHs worked on xyloglucan-cellulose network, modified the cell wall via enzymatic mechanisms (Nishitani & Tominaga, 1992; Rose et al., 2002) and were required during plant growth in cell wall modification (Campbell & Braam, 1999; Rose et al., 2002). It was confirmed that XTHs were necessary in cell wall restructuring during cellular expansion, which fueled rapid petiole elongation (Sasidharan et al., 2010). XTH16 was also involved in radish taproot thickening (Yu et al., 2015). *Ghir_A02G001110* was annotated as *IQD13* and was interacting with both microtubules and the plasma membrane. It specifically promoted cortical microtubule rescue, which consequently increased cortical microtubule density (Sugiyama et al., 2017). All above DEGs provided insight into the differences of molecular mechanism during fiber development on transcription level, which would be also beneficial for cotton breeding.

### Hub genes identified by WGCNA may have significantly impact on lint percentage and fiber length

In this study, WGCNA was performed to identify hub genes and modules, which were highly associated with cotton fiber initiation and elongation. To investigate the influences of the hub genes on fiber yield and quality traits during fiber development, multiple comparisons with the previous studies were performed. At 5 DPA, *Ghir_A07G015620*, sharing high protein sequence identification with *Gh_A07G1360*, showed highly correlation with boll weight and seed index traits in Zhang et al.’s (2020) report. In Song et al.’s (2019) study, *Ghir_D05G012040* (*Gh_D05G1139*) identified in the module associated with high-LP line L2 at 0 DPA was locating in the QTL related to lint percentage trait. *Ghir_A08G023930* (*Gh_A08G2014*) and *Ghir_A09G017620* (*Gh_A09G2422*), identified in Fiber brown module and Ovule turquoise module, were detected in FL QTL in Naoumkina et al.’s (2019) report. In Sun et al.’s (2017) and Liu et al.’s (2018) studies, *Ghir_D03G003280* (*Gh_D03G0303*) was reported to have influence on fiber length by GWAS analysis. At 0 DPA, *Ghir_A12G024510* was annotated as 1-aminocyclopropane-1-carboxylate synthase 6 in *G. hirsutum* (*GhACS6*), which was identified as the key enzyme involved in ethylene biosynthesis and was considered critically important for cotton fiber elongation (Wang et al., 2007). Meanwhile, *Ghir_A03G022700* showed highly protein sequence identity with a ubiquitin family protein that could interact with and responds to the degradation of *GbPDF1*. *GbPDF1* was confirmed playing a critical role in cotton fiber development and required in fiber initiation, where *PDF1*-silenced cotton showed retarded fiber initiation and had shorter fibers or lower lint percentage (Deng et al., 2012). It was hub genes result suggested that ethylene is significantly important for cotton fiber development. At 10 DPA, *Ghir_D05G023530* shared high identity with endo-1,3-beta-glucanase, which was reported to be involved in secondary wall synthesis accompanying the deposition of cellulose in growing cotton fiber cells (Shimizu et al., 1997), implying that it might have influence on fiber strength. During fiber development, hub genes played important roles and had impact on fiber yield and quality traits. Therefore, the functions and genetic mechanisms of the hub genes were worthy for further exploring.

## Conclusions

In summary, the two extreme *G. hirsutum* RILs selected from CCRI70 RIL population were conducted transcriptome research on fiber initiation and elongation, aiming to understand the parental source of potential alleles and the differential molecular mechanisms associated with LP and FL. As a result, 249/128, 369/206, 4296/1198 and 3547/2129 up-/down- regulated DEGs were obtained during fiber development at −3, 0, 5 and 10 DPA, respectively. According to TM-1, 239493 genotypic variants were identified and 40522 genes were involved. Based on KEGG enrichment analysis on the DEGs at 0 and 5 DPA, galactose metabolism, auxin signaling pathway and etc. were significant enriched into. By STEM, profile 14 and 18 were considered highly associated with cotton fiber initiation and elongation, of which genes expressed differently were analyzed by gene ontology analysis while genes expressed in common were enriched by KEGG. KEGG analysis revealed that the DEGs were involved in the pathways of ribosome, AGE-RAGE signaling pathway, biosynthesis of unsaturated fatty acids and fatty acid metabolism of profile 14. Genes in profile 18 were enriched into the pathways of metabolic pathways, phagosome, biosynthesis of secondary metabolites, starch and sucrose metabolism and oxidative phosphorylation. Co-expression network analysis by using WGCNA identified 29 hub genes in four fiber developmental time points. *Ghir_A03G022700* was annotated as a ubiquitin family protein that could interact with and responds to the degradation of *GbPDF1*, which was considered being critically important and required in fiber initiation. *Ghir_A12G024510* annotated as *GhACS* was identified as the key enzyme involved in ethylene biosynthesis and was considered critically important for cotton fiber elongation. These findings would provide insights into the molecular mechanism of the fiber development, which would be the genetic basis to improve the yield and fiber quality simultaneously of upland cotton breeding.

##  Supplemental Information

10.7717/peerj.11812/supp-1 Supplemental Information 1 Phenotypic data of two lines in 5 environments

10.7717/peerj.11812/supp-2 Supplemental Information 2 Summary of RNA-seq of the 24 tissue samples

10.7717/peerj.11812/supp-3 Supplemental Information 3 The Pearson correlationship coefficient among each sample

10.7717/peerj.11812/supp-4 Supplemental Information 4 The differentially expressed genes (DEGs) of each pair-wise comparison of L1 vs L2 in −3, 0, 5, 10 DPA

10.7717/peerj.11812/supp-5 Supplemental Information 5 The differentially expressed genes (DEGs) of each pair-wise comparison of L1 and L2 between different stages

10.7717/peerj.11812/supp-6 Supplemental Information 6 CD sequences of DEGs

10.7717/peerj.11812/supp-7 Supplemental Information 7 FPKMs and functional categories of genes significantly upregulated and downregulated in L2 on 0 DPA

10.7717/peerj.11812/supp-8 Supplemental Information 8 FPKMs and functional categories of genes significantly upregulated and downregulated in L2 on 5 DPA

10.7717/peerj.11812/supp-9 Supplemental Information 9 SNP variant annotation

10.7717/peerj.11812/supp-10 Supplemental Information 10 The profile lists by STEM analysis

10.7717/peerj.11812/supp-11 Supplemental Information 11 KEGG enrichment analysis and GO annotation result

10.7717/peerj.11812/supp-12 Supplemental Information 12 Genes listed in the modules associated with specific stages

10.7717/peerj.11812/supp-13 Supplemental Information 13 Primers in expression pattern validation

10.7717/peerj.11812/supp-14 Supplemental Information 14 QPCR of expression pattern validation

10.7717/peerj.11812/supp-15 Supplemental Information 15 Variants on the genome

## Additional Information and Declarations

### Data Availability

The following information was supplied regarding data availability: The raw data is available at the National Genomics Data Center (NGDC): CRA002982.
