# Supplementary figures and images for: Co-expression network and comparative transcriptome analysis for fiber initiation and elongation reveal genetic differences in two lines from upland cotton CCRI70 RIL population
Source: PeerJ. 2021 Jul 21;9:e11812. doi: 10.7717/peerj.11812 (PMC8308610; doi:10.7717/peerj.11812)

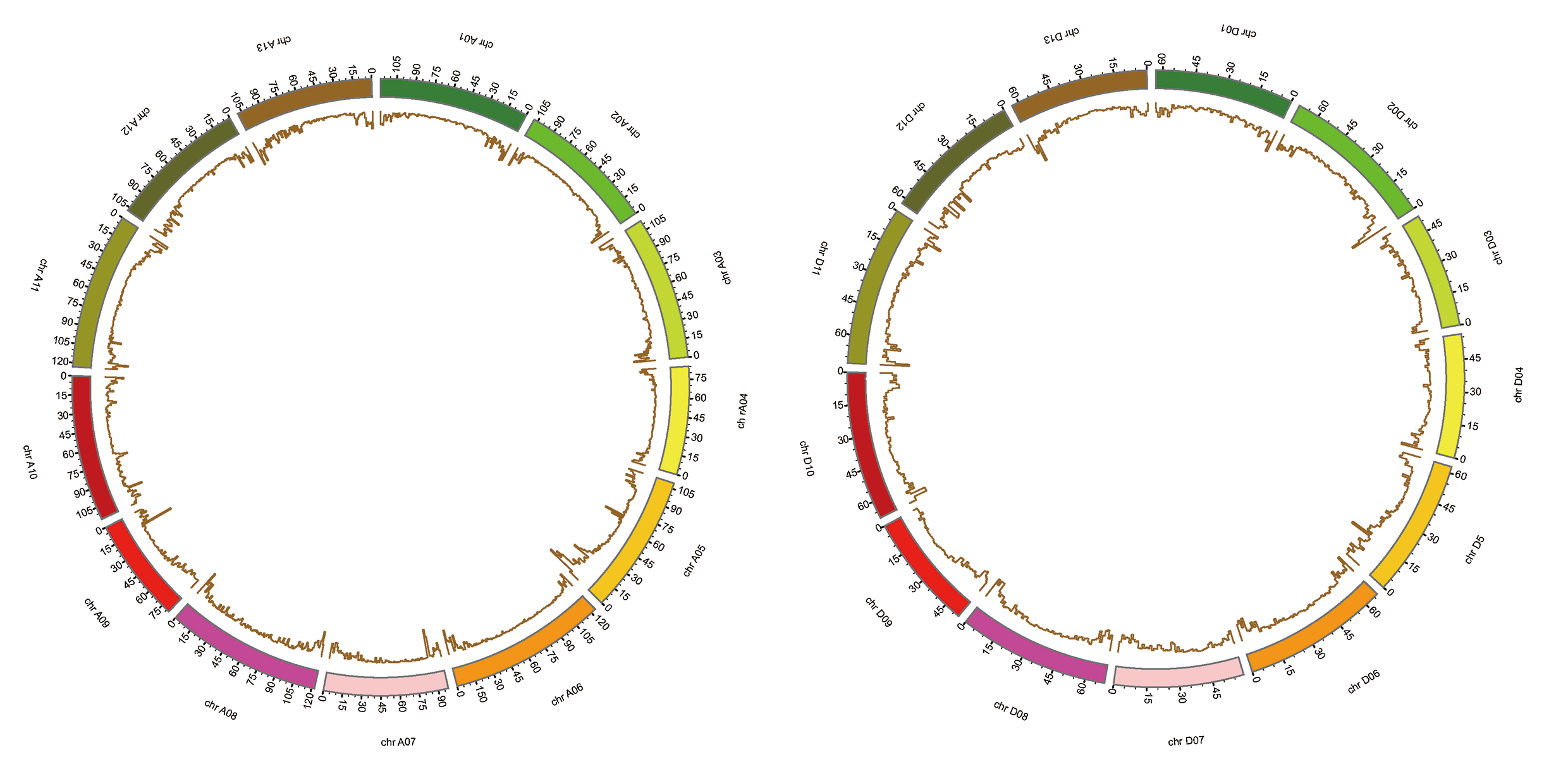

Supplement: Supplemental Information 15 [file peerj-09-11812-s015.png]
